# Efficacy of Hydroxyurea in Transfusion-Dependent Major β-Thalassemia Patients: A Meta-Analysis

**DOI:** 10.7759/cureus.38135

**Published:** 2023-04-26

**Authors:** Modather I Hatamleh, Venkata Sai Harshabhargav Chenna, Hazel Contractor, Gautham Varun Krishna Mohan, Gayathri Tirumandyam, Nada Dammas, Muhammad Waqas Khan, Shamsha Hirani

**Affiliations:** 1 Internal Medicine, King Abdullah University Hospital, Amman, JOR; 2 Medicine, University of Perpetual Help System Dalta, Las Piñas, PHL; 3 Medical Education, Smt. Nathiba Hargovandas Lakhmichand (NHL) Municipal Medical College, Ahmedabad, IND; 4 Internal Medicine, Tirunelveli Medical College, Tirunelveli, IND; 5 Internal Medicine, Siddhartha Medical College, Dr. NTR University of Health Sciences, Vijaywada, IND; 6 Pediatrics, King Faisal Specialist Hospital and Research Centre, Riyadh, SAU; 7 Medicine, Services Institute of Medical Sciences, Lahore, PAK; 8 Cardiology, Baqai Hospital, Karachi, PAK

**Keywords:** meta-analysis, transfusion, efficacy, hydroxyurea, transfusion-dependent β-thalassemia patient

## Abstract

The present meta-analysis was conducted to determine the efficacy of hydroxyurea in patients with transfusion dependent major β-thalassemia. The present meta-analysis was conducted following the Preferred Reporting Items for Systematic Reviews and Meta-Analyses (PRISMA) and Meta-analyses of Observational Studies in Epidemiology (MOOSE) guidelines. A systematic search was carried out to evaluate the efficacy of hydroxyurea in patients with transfusion-dependent B-thalassaemia using electronic databases, including MEDLINE, Cochrane Central Register of Controlled Trials, and EMBASE. The keywords used to search for relevant studies included “hydroxyurea”, “thalassemia”, “transfusion-dependent”, and “efficacy”. Outcomes assessed in the present meta-analysis included transfusion in one year and intervals between transfusions (in days). Other outcomes assessed in the present meta-analysis were fetal hemoglobin (%), hemoglobin (%), and ferritin levels (ng/dl). Total of five studies were included in the analysis enrolling 294 patients with major B-thalassemia. The pooled analysis reported that the mean interval between transfusions was significantly higher in patients receiving hydroxyurea compared to those not receiving hydroxyurea (mean deviation {MD}: 10.07, 95% CI: 2.16, 17.99). Hemoglobin was significantly higher in patients receiving hydroxyurea compared to its counterparts (MD: 1.71, 95% CI: 0.84, 2.57). Patients receiving hydroxyurea had significantly lower ferritin levels compared to those not receiving hydroxyurea (MD: -299.65, 95% CI: -518.35, -80.96). These findings suggest that hydroxyurea may be a promising and cost-effective alternative to blood transfusions and iron chelation therapies for beta-thalassemia patients. However, the authors noted that further randomized controlled trials are needed to validate these findings and to determine the optimal dosages and treatment regimens for hydroxyurea in this patient population.

## Introduction and background

One of the most common inherited illnesses worldwide is β-thalassemia, characterized by a decreased ability to generate hemoglobin [1]. β-thalassemia is divided into two groups, including non-transfusion-dependent and transfusion-dependent β-thalassaemia [2]. Patients with transfusion-dependent β-thalassemia include β-thalassemia severe and major hemoglobin, and it requires regular blood transfusions every two to five weeks for life [3].

Currently, allogenic hematopoietic stem cell transplantation is the only known cure for β-thalassemia. However, it is limited to a small number of patients due to donor availability and cost constraints [4]. Different novel treatment options that include genome editing and gene therapy have shown promise in clinical and preclinical studies [5-6]. However, none of these treatments have reached the level of being considered standard patient care as of now. As a result, most patients with transfusion-dependent β-thalassemia only receive supportive treatment in the form of regular blood transfusions throughout their lives [7]. Regular blood transfusions lead to iron overload, resulting in organ damage and, ultimately, death of these patients [8]. Therefore, there is a need for alternative therapy to reduce the burden of blood transfusions in patients with transfusion-dependent β-thalassemia.

Hydroxyurea (or hydroxycarbamide), primarily a cytotoxic, anti-metabolic, and antineoplastic agent, also induces fetal hemoglobin (HbF) synthesis by stimulating γ-globin production. Besides stress erythropoiesis, which is considered to be the primary mechanism, production of nitric oxide and the soluble guanylyl cyclase and cyclic guanosine monophosphate-dependent protein kinase pathway gene have been proposed as being responsible for inducing γ-globin synthesis [9]. Apart from its established role in stimulating γ-globin production, hydroxyurea may also have a broader impact in enhancing globin synthesis, including β-globin, in certain patients who are able to express normal β-globin chains [10]. Therefore, hydroxyurea induces not only hemoglobin F but also overall hemoglobin production. After being identified as a potent hemoglobin inducer, hydroxyurea became one of the important therapeutic agents for the management of patients with sickle cell anemia and has been widely assessed in thalassemia intermedia, with varying results [11].

Despite being utilized for individuals with non-transfusion-dependent β-thalassemia, the effectiveness of hydroxyurea in transfusion-dependent β-thalassemia remains uncertain due to the absence of properly designed clinical trials. The limited numbers of published studies that have examined the effects of hydroxyurea in this patient population have yielded conflicting results, with varying rates of response [12-13]. Therefore, the efficacy of hydroxyurea in transfusion-dependent β-thalassemia is not well-established due to the lack of robust clinical evidence. Hence, the present meta-analysis has been conducted to determine the efficacy of hydroxyurea in patients with transfusion-dependent major β-thalassemia.

## Review

Methodology

The present meta-analysis was conducted following the Preferred Reporting Items for Systematic Reviews and Meta-Analyses (PRISMA) and Meta-analyses of Observational Studies in Epidemiology (MOOSE) guidelines.

Data Sources and Searches

A systematic search was carried out to evaluate the efficacy of hydroxyurea (HU) in patients with transfusion-dependent B-thalassaemia using electronic databases including MEDLINE, Cochrane Central Register of Controlled Trials, and EMBASE. The keywords used to search for relevant studies included “hydroxyurea”, “thalassemia”, “transfusion-dependent”, and “efficacy”. Medical subject headings (MeSH) terms and Boolean operators were also used to conduct the search.

All relevant studies were independently assessed by two authors. After removing duplicates, initial screening was done using titles and abstracts. The full-text of eligible articles was retrieved, and a detailed evaluation of pre-defined inclusion and exclusion criteria was conducted. Any disagreement in the process of searching and study selection was resolved via discussion.

Eligibility Criteria

We included randomized controlled trials (RCTs), observational studies, pre-post studies, and quasi-experimental studies that assessed the efficacy of hydroxyurea alone in patients with transfusion-dependent major β-thalassaemia. We excluded studies that included non-transfusion-dependent β-thalassaemia or intermedia β-thalassemia. We also excluded studies with any combination therapy with hydroxyurea, case reports, case series, and review articles. Pre-post studies with different follow-up periods in pre-intervention and post-intervention periods were excluded from the present meta-analysis. We also excluded studies that did not report desired outcomes.

Data Abstraction and Outcome Measures

Two reviewers extracted the data independently using the data extraction form developed using Microsoft Excel. Key characteristics were extracted from the included studies and records, including author name, year of publication, study design, region where the study was conducted, sample size, dose of hydroxyurea, follow-up duration, and outcome measures. Outcomes assessed in the present meta-analysis included transfusion in one year and intervals between transfusions (in days). Other outcomes assessed in the present meta-analysis were fetal hemoglobin (%), hemoglobin (%), and ferritin levels (ng/dl).

Quality Assessment

The quality assessment of the included studies were done using the National Institutes of Health (NIH) quality assessment tool. The tool is composed of 12 questions assessing risk for selection bias, information bias, measurement bias, and confounding each study was judghed to be of “good,” “fair,” or “poor” quality. 

Data Synthesis and Analysis

For continuous outcomes, mean difference (MD) with their 95% confidence intervals (CIs) were reported. A p-value less than 0.05 was considered significant. Heterogeneity was reported among the study results using I-square statistics and the Cochran's Q test. For an I-square value of <50%, a fixed-effect model was used; otherwise, a random effect model was used. For the Cochran's Q test, a cut-off p-value of 0.1 was used. Forest plots were used to present pooled estimates of outcomes assessed in the present meta-analysis. The analysis was carried out using Review Manager Version 5.4.1 (The Cochrane Collaboration, London, United Kingdom).

Results

**Figure 1:**
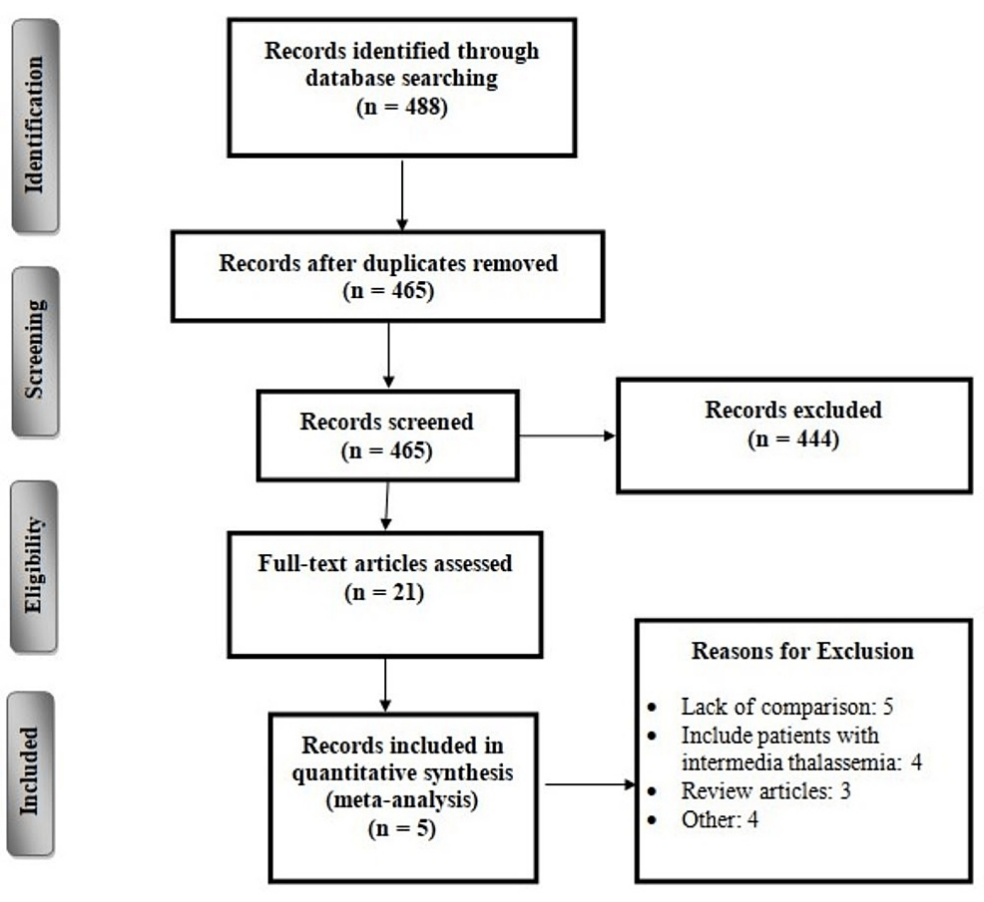
Flowchart of selection of studies

The initial database search identified 488 references. After removing duplicates, 465 studies were assessed for eligibility criteria using titles and abstracts. Full texts of 21 articles were obtained, and a detailed assessment of inclusion and exclusion criteria was done. Of these 21 articles, five studies met the inclusion and exclusion criteria and were included in the present meta-analysis. Figure 1 shows the flowchart of study selection.

Of these seven studies, one was a randomized control trial, one was quasi-experimental, while the remaining were pre-post interventional studies. In all included studies, hydroxyurea was used as a single intervention with no other treatment apart from blood transfusion. In all studies, hydroxyurea was given as a single oral agent, and the dose of the drug was similar across all the included studies, ranging from 10 mg/kg/day to 20 mg/kg/day. In pre-post studies, the pre-study arm was used as a historical control, and the post-study arm was utilized as the treatment group, where hydroxyurea was given. All included studies were published in the English language and as full-text papers. The studies collectively enrolled 294 patients with major B-thalassemia. The sample size of individual studies ranged from n=12 to n=100. Study populations were a mixture of children and adults in all studies. The study characteristics are shown in Table 1. Table 2 shows quality assessment of included studies.

**Table 1:** Characteristics of included studies RCT: Randomized-controlled trial

Author Name	Year	Region	Design	Participants	Sample Size	Dose of hydroxyurea	Follow-up
Akram et al. [14]	2022	Pakistan	Quasi-experimental	Pediatric cases of beta-thalassemia major aged between five years and 18 years that were on regular blood transfusions	100	10–20 mg/kg/ day	12 Months
Bordbar et al. [15]	2014	Iran	Pre-post	Patients with transfusion-dependent β-thalassaemia aged over 2 years	97	10.5 mg/kg/ day	14 months
Suthar et al. [16]	2017	India	Pre-post	Patients with transfusion-dependent β-thalassaemia aged between 2 and 18 years	12	20 mg/kg/ day	6 Months
Yadav et al. [17]	2016	India	Pre-post	Patients with thalassemia major aged 3 years to 18 years	25	10–20 mg/kg/ day	12 Months
Yasara et al. [18]	2022	Srilanka	RCT	Patients with transfusion-dependent β-thalassaemia aged over 12 years and required more than eight blood transfusions during the proceeding one year were eligible to participate in the study	60	10–20 mg/kg/ day	6 Months

**Table 2:** Quality assessment of included studies CD: Cannot be defined

Criteria		Akram et al. [14]	Bordbar et al. [15]	Suthar et al. [16]	Yadav et al. [17]	Yasara et al. [18]
1	Was the study question or objective clearly stated?	Yes	Yes	Yes	Yes	Yes
2	Were eligibility/selection criteria for the study population prespecified and clearly described	Yes	Yes	Yes	Yes	Yes
3	Were the participants in the study representative of those who would be eligible for the test/service/intervention in the general or clinical population of interest?	Yes	Yes	Yes	Yes	Yes
4	Were all eligible participants that met the prespecified entry criteria enrolled?	CD	Yes	Yes	CD	CD
5	Was the sample size sufficiently large to provide confidence in the findings?	No	Yes	No	No	No
6	Was the test/service/intervention clearly described and delivered consistently across the study population?	Yes	Yes	Yes	Yes	Yes
7	Were the outcome measures prespecified, clearly defined, valid, reliable, and assessed consistently across all study participants?	Yes	Yes	Yes	Yes	Yes
8	Were the people assessing the outcomes blinded to the participants’exposures/interventions?	No	No	Yes	No	No
9	Was the loss to follow-up after baseline 20% or less? Were those lost to follow-up accounted for in the analysis?	Yes	Yes	No	Yes	Yes
10	Did the statistical methods examine changes in outcome measures from before to after the intervention? Were statistical tests done that provided p values for the pre-to-post changes?	Yes	Yes	Yes	Yes	Yes
11	Were outcome measures of interest taken multiple times before the intervention and multiple times after the intervention (ie did they use an interrupted time-series design)?	Yes	Yes	Yes	Yes	Yes
12	If the intervention was conducted at a group level (eg a whole hospital, a community, etc.) did the statistical analysis take into account the use of individual-level data to determine effects at the group level?	Yes	Yes	Yes	Yes	Yes
Overall		Good	Fair	Fair	Fair	Good

Meta-Analysis of Outcomes

**Figure 2:**
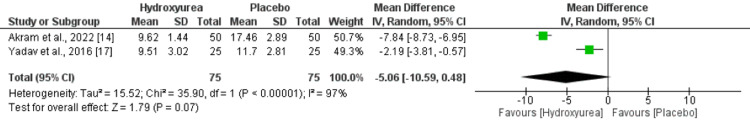
Forest plot showing effect of hydroxyurea on number of blood transfusion Sources: References [14,17]

**Figure 3:**
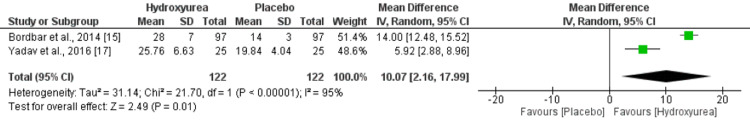
Forest plot showing effect of hydroxyurea on interval between blood transfusion Sources: References [14,17]

Two studies compared the number of transfusions in two groups. The mean number of transfusions was lower in patients receiving hydroxyurea compared to those not receiving hydroxyurea. However, the difference was statistically insignificant (mean deviation {MD}: -5.06, 95% CI: -10.59, 0.48), as shown in Figure 2. High heterogeneity was reported among the study results. Regarding transfusion interval in days, two studies assessed the outcome. The pooled analysis reported that the mean interval between transfusions was significantly higher in patients receiving hydroxyurea compared to those not receiving hydroxyurea (MD: 10.07, 95% CI: 2.16, 17.99), as shown in Figure 3. High heterogeneity was reported among the study results.

**Figure 4:**
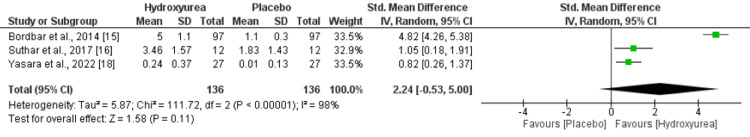
Forest plot showing effect of hydroxyurea on HbF Sources: References [15-16,18]

Three studies compared the change in HbF between two groups. Pooled meta-analysis showed that the mean HbF was higher in patients receiving hydroxyurea; however, the difference was statistically insignificant (MD: 2.24, 95% CI: -0.53, 5.00), as shown in Figure 4.

**Figure 5:**
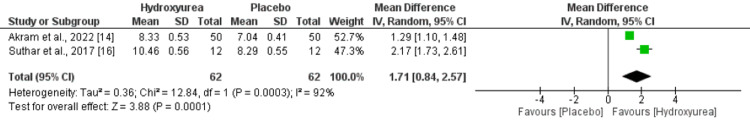
Forest plot showing effect of hydroxyurea on Hemoglobin Sources: References [14,16]

High heterogeneity was reported among the study results. Two studies compared hemoglobin levels. Hemoglobin was significantly higher in patients receiving hydroxyurea compared to its counterparts (MD: 1.71, 95% CI: 0.84, 2.57), as shown in Figure 5.

**Figure 6:**
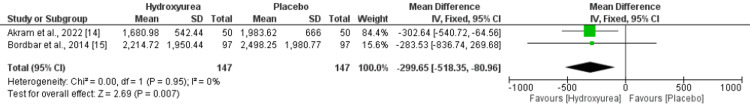
Forest plot showing effect of hydroxyurea on ferritin Sources: References [14-15]

High heterogeneity was reported among the study results. Two studies compared ferritin levels. Patients receiving hydroxyurea had significantly lower ferritin levels compared to those not receiving hydroxyurea (MD: -299.65, 95% CI: -518.35, -80.96), as shown in Figure 6. No heterogeneity was reported among the study results.

Discussion

This meta-analysis evaluates the clinical efficacy of hydroxyurea in transfusion-dependent B-thalassemia. Five studies met our inclusion criteria, and most were observational pre-post studies. Our meta-analysis concluded that hydroxyurea has good clinical efficacy in increasing hemoglobin and decreasing transfusion requirements in individuals with transfusion-dependent β-thalassemia. The meta-analysis conducted by Algiraigri et al. for non-transfusion-dependent β-thalassemia found that hydroxyurea had good clinical efficacy in decreasing transfusion requirements and increasing hemoglobin levels [19].

Regular blood transfusions and the use of iron chelators are still the primary treatment methods for major β-thalassemia. However, there is great potential for pharmacologic reactivation of γ-globin genes in the treatment of thalassemia syndromes and sickle cell disease. Hydroxyurea has been shown to increase γ-chain synthesis and HbF production, and it has been successfully used to treat sickle cell anemia by raising HbF levels and reducing clinical complications. However, limited knowledge exists on the effectiveness of this drug in beta-thalassemic patients [20-21].

Reduction in blood transfusion is a significant benefit for patients with β-thalassemia that is associated with a decrease in short-term and long-term complications and risks of blood transfusion. In particular, this leads to a decrease in iron overload and related end-organ failure and complications, along with less frequent clinical visits. Regular blood transfusions are also associated with a significant financial burden, especially in developing nations. On the other hand, hydroxyurea is an economical drug [22]. More widespread use of hydroxyurea in thalassemia could potentially reduce the indirect and direct costs of transfusions along with iron chelation therapies significantly [19]. An RCT conducted by Yasara et al. found that hydroxyurea continues to need a low volume of blood volumes even during the post-treatment period. This is the only RCT that explored the efficacy of hydroxyurea in patients with transfusion-dependent B-thalassemia [18].

The study conducted by Fucarosen et al. reported the effects of orally administered HU in 13 patients diagnosed with beta-thalassemia major/HbE. The results showed that almost all patients responded positively to the oral dose of HU (10-20mg/kg/d) for a period of five months. They observed a slight increase (10%) in hemoglobin levels, which was statistically significant. Additionally, there was an improvement in the balance between α and non-α globin chain ratios [23]. The notable reduction of serum ferritin levels observed in the current meta-analysis is of great clinical significance since iron overload poses a significant risk to these individuals. The reduction in serum ferritin is mainly attributed to a decrease in blood transfusions and, to a lesser extent, an increase in iron utilization resulting from an increase in hemoglobin production and a reduction in ineffective erythropoiesis.

Study limitations

The present meta-analysis has certain limitations. Firstly, only one RCT was conducted and large numbers of studies were having pre-post interventional studies. Therefore, future studies need to be conducted to warrant these findings. Secondly, there was relatively shorter follow-up time period for the hydroxyurea treatment (an average of 12 months to 14 months in majority of the studies) which limits conclusions regarding the long-term efficacy. Although there are limitations and a need for more rigorous experimental studies, the findings of this meta-analysis indicate that hydroxyurea therapy may have the potential to be effective in patients with transfusion dependent B-thalassemia patients. Therefore, after careful discussions with patients and their families and implementing a monitoring plan for safety and effectiveness, we suggest trials of hydroxyurea therapy for patients with transfusion dependent B-thalassemia patients based on the results of this meta-analysis.

## Conclusions

The meta-analysis included a total of five studies that investigated the effects of hydroxyurea on patients with beta-thalassemia. The results of the analysis showed that hydroxyurea treatment was associated with increased hemoglobin levels and decreased transfusion requirements in these patients. Additionally, patients who received hydroxyurea had significantly lower ferritin levels compared to those who did not receive the treatment. These findings suggest that hydroxyurea may be a promising and cost-effective alternative to blood transfusions and iron chelation therapies for beta-thalassemia patients. However, the authors noted that further randomized controlled trials are needed to validate these findings and to determine the optimal dosages and treatment regimens for hydroxyurea in this patient population.
